# Molecular investigation and phylogeny of *Anaplasmataceae* species infecting domestic animals and ticks in Corsica, France

**DOI:** 10.1186/s13071-017-2233-2

**Published:** 2017-06-23

**Authors:** Mustapha Dahmani, Bernard Davoust, Djamel Tahir, Didier Raoult, Florence Fenollar, Oleg Mediannikov

**Affiliations:** Aix Marseille Univ, CNRS, IRD, INSERM, AP-HM, URMITE, IHU - Méditerranée Infection, Marseille, France

**Keywords:** Corsica Island, Animals, Ticks, *Anaplasma ovis*, *Anaplasma marginale*, *Anaplasma* sp., *Ehrlichia canis*

## Abstract

**Backgrounds:**

Corsica is a French island situated in the Mediterranean Sea. The island provides suitable natural conditions to study disease ecology, especially tick-borne diseases and emerging diseases in animals and ticks. The family *Anaplasmataceae* is a member of the order *Rickettsiales*; it includes the genera *Anaplasma*, *Ehrlichia*, *Neorickettsia* and *Wolbachia*. Anaplasmosis and ehrlichiosis traditionally refer to diseases caused by obligate intracellular bacteria of the genera *Anaplasma* and *Ehrlichia*. The aim of this study was to identify and estimate the prevalence of *Anaplasmataceae* species infecting domestic animals and ticks in Corsica.

**Methods:**

In this study, 458 blood samples from sheep, cattle, horses, goats, dogs, and 123 ticks removed from cattle, were collected in Corsica. Quantitative real-time PCR screening and genetic characterisation of *Anaplasmataceae* bacteria were based on the 23S rRNA, *rpoB* and *groEl* genes.

**Results:**

Two tick species were collected in the present study: *Rhipicephalus bursa* (118) and *Hyalomma marginatum marginatum* (5). Molecular investigation showed that 32.1% (147/458) of blood samples were positive for *Anaplasmataceae* infection. *Anaplasma ovis* was identified in 42.3% (93/220) of sheep. *Anaplasma marginale* was amplified from 100% (12/12) of cattle and two *R. bursa* (2/123). Several potentially new species were also identified: *Anaplasma* cf. *ovis*, “*Candidatus* Anaplasma corsicanum”, “*Candidatus* Anaplasma mediterraneum” were amplified from 17.3% (38/220) of sheep, and *Anaplasma* sp. marginale-like was amplified from 80% (4/5) of goats. Finally, one *R. bursa* tick was found to harbour the DNA of *E. canis*. All samples from horses and dogs were negative for *Anaplasmataceae* infection.

**Conclusions:**

To our knowledge, this study is the first epidemiological survey on *Anaplasmataceae* species infecting animals and ticks in Corsica and contributes toward the identification of current *Anaplasmataceae* species circulating in Corsica.

## Background

Bacteria from the genera *Anaplasma* and *Ehrlichia* are obligate intracellular bacteria transmitted by arthropods, mainly ticks, from one vertebrate host to another. Transmission usually occurs transstadially [1, 2], although transovarial transmission has been reported [3]. In the vertebrate host, the bacteria infect hematopoietic cells [4, 5]. *Anaplasma* and *Ehrlichia* can cause a persistent infection in vertebrate hosts, which allows these hosts to be reservoirs [1, 5]. Probable cases of *Anaplasmataceae* infection in domestic animals were known as early as the beginning of the twentieth century. However, wide interest in studying these bacteria arose when discovering species pathogenic for humans [1]. *Anaplasma phagocytophilum* was known to cause disease in domestic ruminants in Europe and the USA decades before its identification in humans [6]. The first European case of human anaplasmosis was reported in 1995 in Slovenia; after that, human cases have been reported in many countries of Europe [7–11]. Bovine anaplasmosis due to *A. marginale* results in the development of mild to severe anaemia and occurs in tropical and subtropical regions, including South and Central America, the United States, southern Europe, Africa, Asia and Australia [12]. In India, mortality due to bovine anaplasmosis is estimated at between 5 and 40% but may reach up to 70% during a severe outbreak [13]. The economic loss due to infections caused by *Babesia* and *Anaplasma* infections in India was estimated to be $57 million [14]. In Europe, *A. marginale* has spread up to the northern latitudes of Switzerland, Austria and Hungary [15]. *Anaplasma centrale*, a less pathogenic organism but closely related to *A. marginale*, was reported in cattle in Sicily, Italy [16], and from roe deer in Spain [17]. *Anaplasma ovis* is an intraerythrocytic pathogen of sheep, goats and wild ruminants [18]. It is thought to cause only mild clinical symptoms, thus being of minor economic importance [19]. Ovine anaplasmosis appears to be widespread and found in different regions of the world. The extent of the infection and the loss of livestock productivity remain poorly understood [19]. The historical record of this bacterium in Europe was established in Russia in 1929 and 1930 by Yakimoff et al. [19], and in France by Cuille et al. in 1935 and 1936 [19]. In 2007, *A. ovis* human infection was reported in a 27-year-old woman in Cyprus [20].

The management of vector-borne diseases requires increased communication between physicians and veterinarians, particularly when physicians are dealing with patients with unexplained febrile illnesses in an endemic area were pathogen like *Anaplasmataceae* largely interconnected in an epidemiological network involving animals, vectors and humans [21]. Corsica is a French island in the Mediterranean Sea close to the south-east French coast, Sardinia, and the west Italian coast. Highly endemic flora and fauna and endemic pathologies are characteristic in Corsica [22]. Recently, we reported the emergence of Toscana virus in dogs in this region [23] and West Nile virus in domestic animals [24]. Our main objective was to continue the epidemiologic investigation of neglected infectious diseases in animals. To date, the occurrence of *Anaplasmataceae* bacteria in Corsica in domestic animals has never been reported. The aim of this study was to screen for the presence and the prevalence of *Anaplasmataceae* species infecting and currently circulating in domestic animals and their ticks in this region.

## Methods

### Sampling

**Fig. 1:**
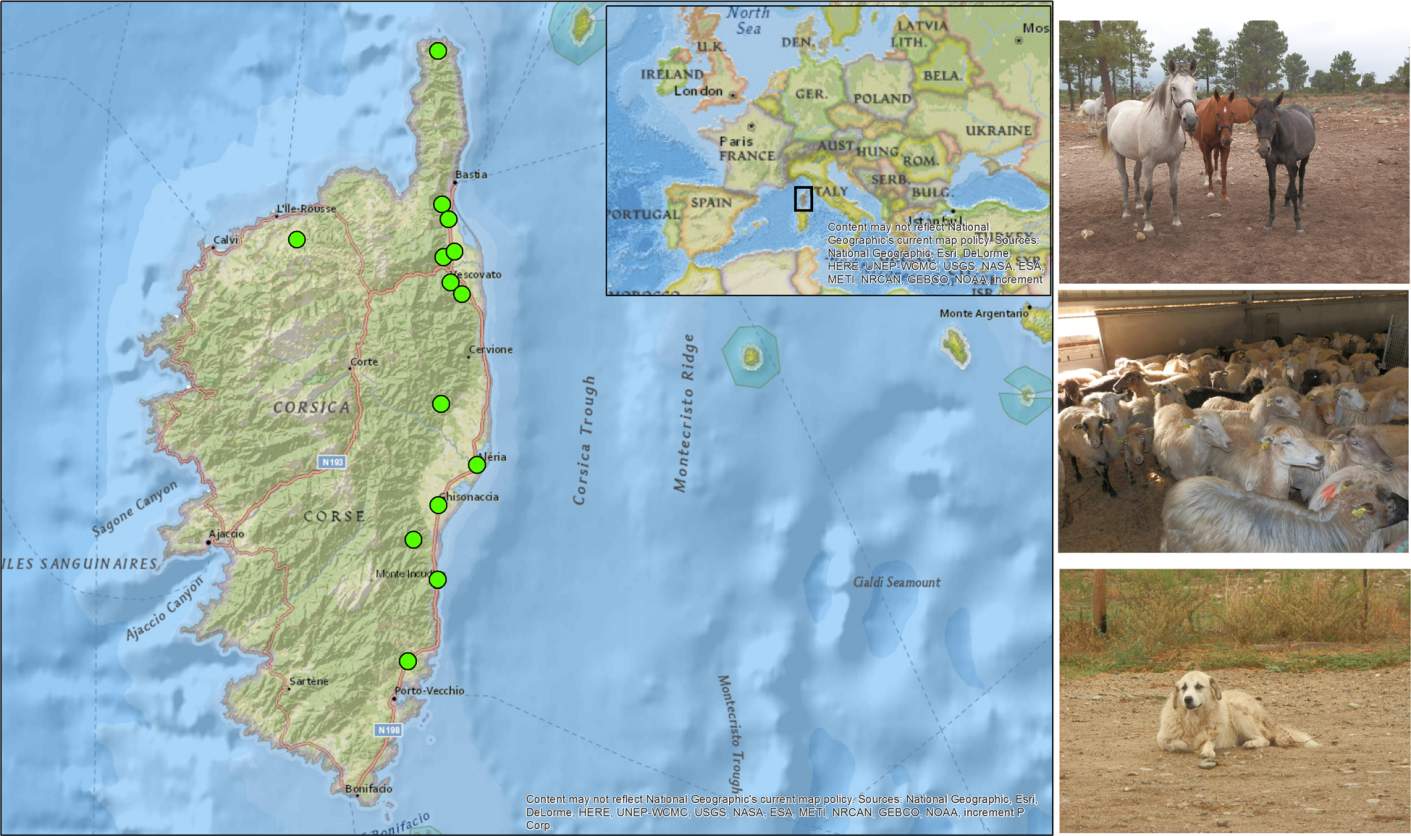
Map of Corsica, France, showing the study areas where the animals were sampled

From 2014 to 2015, EDTA blood samples were obtained from domestic animals on different farms from 14 different areas situated on the east coast of Corsica, France (Fig. 1). Sheep and goats were sampled on the Aléria plain. Cattle and ticks were sampled from one farm in Centu Mezzini, Balagne (42°34′58.242″N, 8°58′38.015″E), whereas dogs and horses were sampled in different localities along the east coast of Corsica island, including Cap Corse (42°56′44″N, 9°26′28″E), Furiani (42°3932″N, 9°24′54″E), Biguglia (42°37′41″N, 9°25′14″E), Lucciana (42°32′48″N, 9°25′5″E), Vescovato (42°29′41″N, 9°26′26″E), Castellare (42°28′7″N, 9°28′27″E), Tallone (42°13′55″N, 9°24′53″E), Ghisonaccia (42°1′3″N, 9°24′20″E), Solenzara (41°55′36″N, 9°24′19″E), Lecci (41°40′48″N, 9°19′5″E), Borgo (42°33′17″N, 9°25′41″E), and Ventiseri-Solenzara (41°55′36″N, 9°24′19″E) (Table 1). Sheep blood samples (230) were collected from three farms. In two farms, the sheep appeared healthy; however, the farmers declared that their sheep experienced many health problems during the winter of 2014, including respiratory disorders and a drop in milk production. At the third sheep farm, the farmer declared that the sheep at his farm were currently unhealthy, with a variety of symptoms, including recurrent fever, abortion, and some sheep died. A cattle herd in Balagne consisted of 16 cows. The cows in this herd had pronounced anaemia with icterus, and some of them died in 2015. Goats (*n* = 5) were all sampled on one farm; they had anaemia and a drop in milk production. In addition, blood samples were collected from horses at a different ranch. Dogs sampled in the present study included hunting dogs, sheep dogs, military working dogs and some pet dogs. Animals were examined with the assistance of their owners. Blood samples were collected by a veterinarian. After transport to the laboratory in Marseille, all samples were stored at -80 °C.

**Table 1:** Origin of animal and tick samples collected and investigated in this study

Species	Number	Origin	Tick infestation	No. of ticks
2014				
Sheep	201	Aléria Plain	not found	–
Horse	98	East coast	not found	–
Dog	73	East coast	not found	–
2015				–
Sheep	19	Aléria Plain	not found	–
Cattle	12	Balagne	*R. bursa*	118
			*Hy. m. marginatum*	5
Goat	5	Aléria Plain	not found	–
Dog	50		not found	–
Totals	458			123

### Tick collection and identification

From the cattle farm, ticks were collected manually from adult cattle and stored in 70% ethanol until identification. Morphological identification was performed with a binocular microscope. Ticks were classified by family, genus and species using available taxonomic keys and morphometric tables [25, 26]. In addition, to confirm the morphological identification, three morphologically identified specimens of each species and all ticks that were not identified, or identified only to the genus level (engorged females and damaged ticks) were subjected to molecular identification using primers targeting the mitochondrial 12S rRNA gene, as previously described [27].

### DNA extraction

DNA extraction was performed on the BioRobot EZ1 (Qiagen, Courtaboeuf, France) using a commercial EZ1 DNA Tissue Kit (Qiagen) according to the manufacturer’s instructions. DNA was extracted from 200 μl of blood from all animal samples. Ticks were recovered from ethanol, rinsed with distilled water and dried on sterile filter paper in a laminar-flow hood. Each tick was cut in half lengthways (the blades were discarded after each tick was cut). DNA was individually extracted from one half, and the remaining halves of the ticks were frozen at -80 °C for subsequent studies, as previously described [28].

### PCR amplification

For the molecular identification of the species of selected ticks, the DNA samples were subjected to standard PCR to amplify a 360-base-pair (bp) fragment of the mitochondrial 12S rRNA gene (Table 2). To investigate the presence of *Anaplasmataceae* in Corsican ticks and domestic animals, DNA from ticks and blood were initially screened by a qPCR targeting the 23S rRNA gene. This qPCR has been reported to amplify most bacteria belonging to the family *Anaplasmataceae* [29]. Then, all positive samples were subjected to conventional PCR using the primers that amplify a 485 bp fragment of the 23S rRNA gene, as previously described [29]. In order to mine deeper into the identification of *Anaplasmataceae* species in domestic animals or ticks, positive samples were tested by PCR using *Anaplasma* genus-specific primers targeting the 525 bp fragment of the RNA polymerase subunit beta (*rpoB*) gene, and *Ehrlichia* genus-specific primers targeting the 590 bp fragment of the heat shock protein (*groEl*) gene [28] (Table 2).

**Table 2:** Primers and probes used in this study

Targeted microorganisms	Targeted gene	Primers and probe^a^	Sequences 5′-3′	Annealing temperature (°C)	References
		qPCR			
*Anaplasmataceae*	23S rRNA	TtAna-F TtAna-R TtAna-S^a^	TGACAGCGTACCTTTTGCAT GTAACAGGTTCGGTCCTCCA FAM-CTTGGTTTCGGGTCTAATCC-TAMRA	60	[28, 29]
		Conventional PCR			
*Anaplasmataceae*	23S rRNA	Ana23S-212f Ana23S-753r	ATAAGCTGCGGGGAGTTGTC TGCAAAAGGTACGCTGTCAC	55	[28, 29]
*Anaplasma* spp.	*rpoB*	Ana-*rpoB*F Ana-*rpoB*R	GCTGTTCCTAGGCTYTCTTACGCGA AATCRAGCCAVGAGCCCCTRTAWGG	55	[27]
*Ehrlichia* spp.	*groEL*	Ehr-*groEL*-F Ehr-*groEL*-R	GTTGAAAARACTGATGGTATGCA ACACGRTCTTTACGYTCYTTAAC	50	[27]
Ticks	12S rRNA	T1B T2A	AAACTAGGATTAGATACCCT AATGAGAGCGACGGGCGATGT	51	[26]

^a^Probe

PCR amplifications were performed as described previously [29, 30]. Briefly, quantitative real-time PCR assays were performed on the CFX96 Touch detection system (Bio-Rad, Marnes-la-Coquette, France) using the Takyon Master Mix according to the manufacturer’s instructions. The conventional PCRs were performed in automated DNA Thermal cyclers (GeneAmp PCR Systems Applied Biosystems, Courtaboeuf, France). The amplification reactions were performed under the following conditions: an initial denaturation step at 95 °C for 15 min, followed by 40 cycles consisting of 1 min denaturation at 95 °C, 1 min annealing at a corresponding temperature (Table 2) and a 1 min extension at 72 °C. A final extension cycle at 72 °C for 7 min was performed and the reactions were cooled at 15 °C. Distilled water was used as negative control. Positive control used was the DNA of *A. phagocytophilum* extracted from the supernatant of the continuous culture of this species in our laboratory, and *E. canis* DNA obtained from infected dogs sampled in Algeria [30]. After electrophoresis, the amplification products were visualised on 1.5% agarose gels stained with ethidium bromide and examined by UV transillumination. A DNA molecular weight marker (marker VI, Boehringer Mannheim, Mannheim, Germany) was used to estimate the size of the products.

### Sequencing and phylogenetic analyses

Sequencing analyses were performed on the Applied Biosystems 3130xl Genetic Analyzer (Thermo Fisher Scientific, France) using the DNA sequencing BigDye Terminator Kit (Perkin-Elmer) according to the manufacturer’s instructions. The obtained sequences were assembled using ChromasPro 1.7 software (Technelysium Pty Ltd., Tewantin, Australia) and the sequences of primers were removed. Sequences obtained in this study were aligned with other ticks or *Anaplasmataceae* species sequences available on GenBank using CLUSTALW implemented on BioEdit v3 [31]. The sequence of 12S rDNA from ticks and the sequences of bacterial 23S rRNA, *rpoB,* and *groEl* genes were first aligned individually, gaps and missing data were eliminated, and then, for the sequences of *Anaplasmataceae* species, the alignment of the 23S rRNA with *rpoB* genes and 23S rRNA with *groEl* gene sequences were concatenated for phylogenetic tree construction for the *Anaplasma* and *Ehrlichia* species, respectively. Phylogenetic and molecular evolutionary analysis were inferred using the maximum likelihood method implemented on MEGA7 [32], with the complete deletion option, based on the Hasegawa-Kishino-Yano (HYK) model for nucleotide sequences. A discrete gamma distribution was used to model evolutionary rate differences among sites. Initial trees for the heuristic search were obtained automatically by applying the neighbor-joining and BIONJ algorithms to a matrix of pairwise distances estimated using the maximum composite likelihood (MCL) approach. Statistical support for internal branches of the trees was evaluated by bootstrapping with 1000 iterations.

## Results

### Tick identification and *Anaplasmataceae* screening

**Fig. 2:**
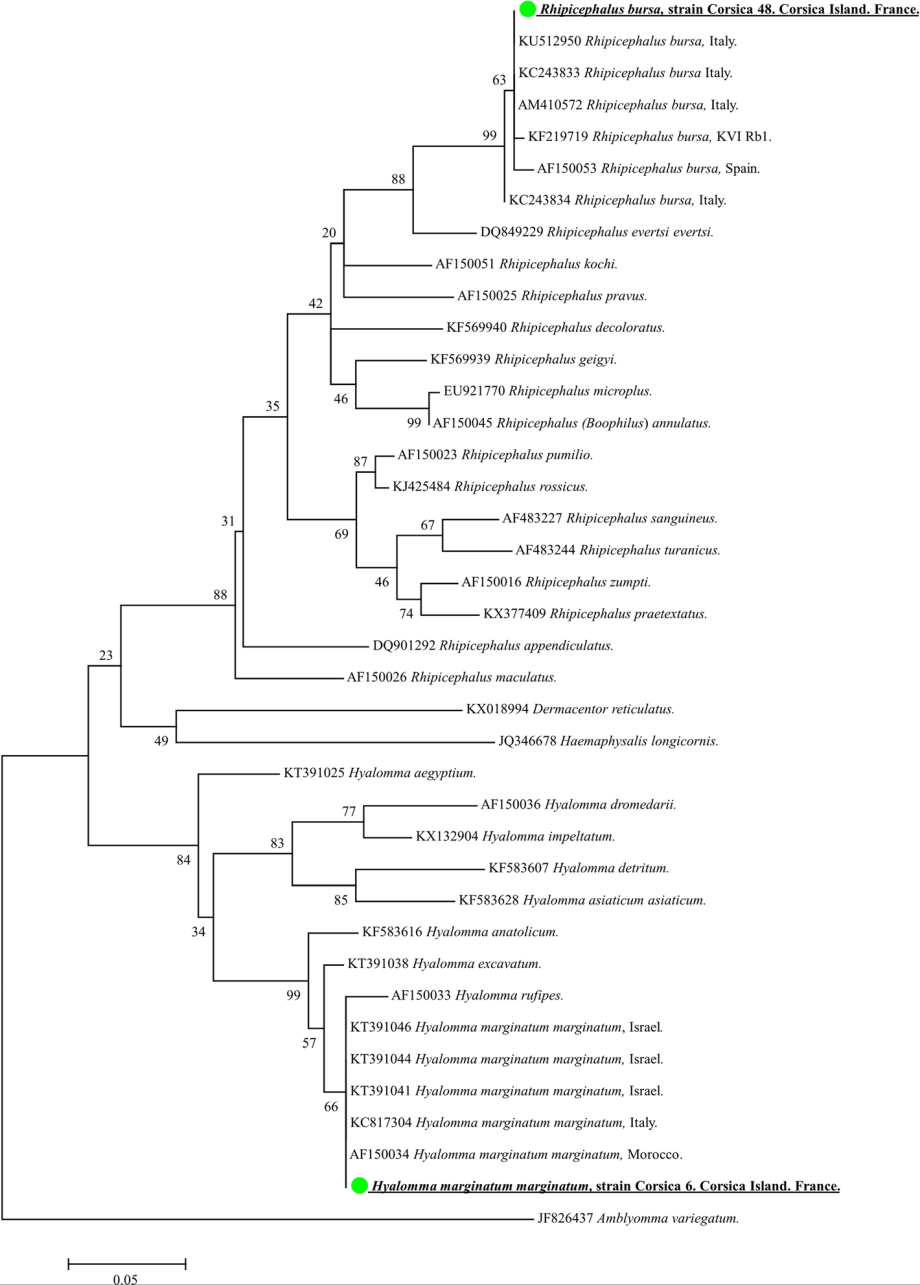
Phylogenetic tree showing the position of *Rhipicephalus bursa* and *Hyalomma marginatum marginatum* compared to other tick species. The evolutionary history was inferred by using the maximum likelihood method based on the Hasegawa-Kishino-Yano model. A discrete Gamma distribution was used to model evolutionary rate differences among sites [4 categories (+G, parameter = 0.2936)]. The analysis involved 39 nucleotide sequences. All positions containing gaps and missing data were eliminated. There was a total of 267 positions in the final dataset. The scale-bar represents a 5% nucleotide sequence divergence

In total, 123 ticks were collected. Eighty-five removed ticks were identified as *Rhipicephalus bursa*, and 3 as *Hyalomma marginatum*. Thirty-five damaged ticks, including 32 engorged ticks, were only morphologically identified to the genus level as follows: 29 ticks *Rhipicephalus* sp., 2 *Hyalomma* sp., and 4 ticks were not identified. Two or three specimens from each tick identified at a species level were selected randomly, and all 35 damaged/engorged ticks were subjected to molecular identification. After 12S rDNA amplification and blast analysis, the six morphologically identified specimens were confirmed to be *R. bursa* and *Hy. marginatum marginatum*. From the 35 damaged ticks, 32 ticks were identified as *R*. *bursa*, and 3 were identified as *Hy. m. marginatum*. All 12S rDNA sequences of the *R. bursa* were identical to each other and showed 100% identity with *R. bursa* from Italy (KU51295, KC243833, AM410572), and 99% identity with *R. bursa* ticks reported from Spain (KC243834) (Fig. 1). All five sequences of *Hy. m. marginatum* were also identical to each other and showed 100% identity with *Hy. m. marginatum* from Italy (KC817304), Israel (KT391046), Morocco (AF150034) and Yemen (HE819515) (Fig. 2). Overall, the ticks collected in this study were as follows: 118 (95.9%) were identified as *R. bursa*; 75 were female, including 30 engorged females, and 43 were male. Five (4.1%) were identified as *Hy. m. marginatum*; two were engorged females, and three were male.

**Fig. 3:**
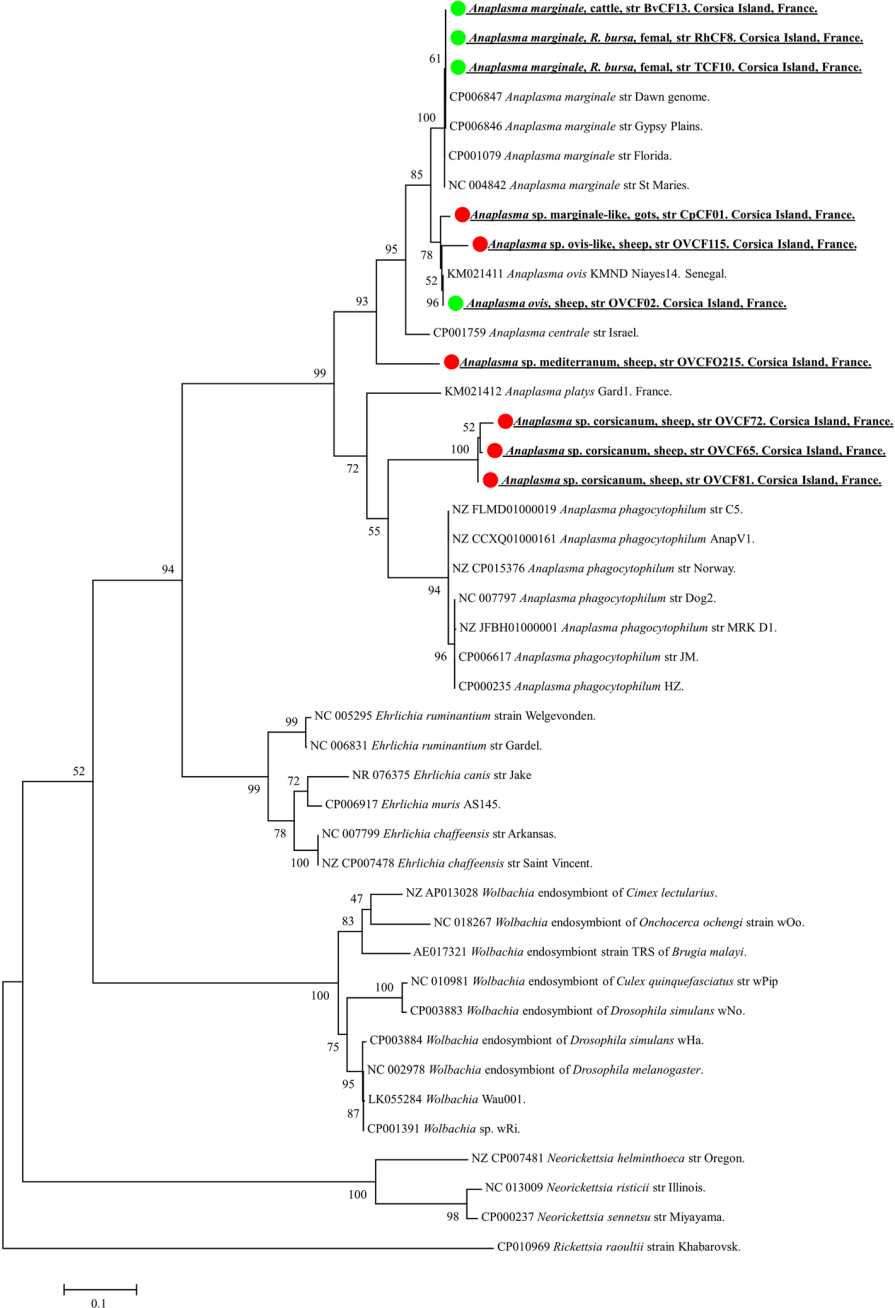
Phylogenetic tree showing the position of *A. marginale* amplified from *R. bursa* and cattle, *A. ovis*, “*Ca*. Anaplasma corsicanum”, *Anaplasma* sp. ovis-like, “*Ca*. Anaplasma mediterraneum” amplified from sheep and *Anaplasma* sp. marginale-like amplified from goats, compared to other species. The evolutionary history was inferred by using the maximum likelihood method based on the Hasegawa-Kishino-Yano model. A discrete Gamma distribution was used to model evolutionary rate differences among sites [2 categories (+G, parameter = 0.3880)]. The analysis involved 43 nucleotide sequences. All positions containing gaps and missing data were eliminated. There was a total of 861 positions in the final dataset. The scale-bar represents a 10% nucleotide sequence divergence

**Fig. 4:**
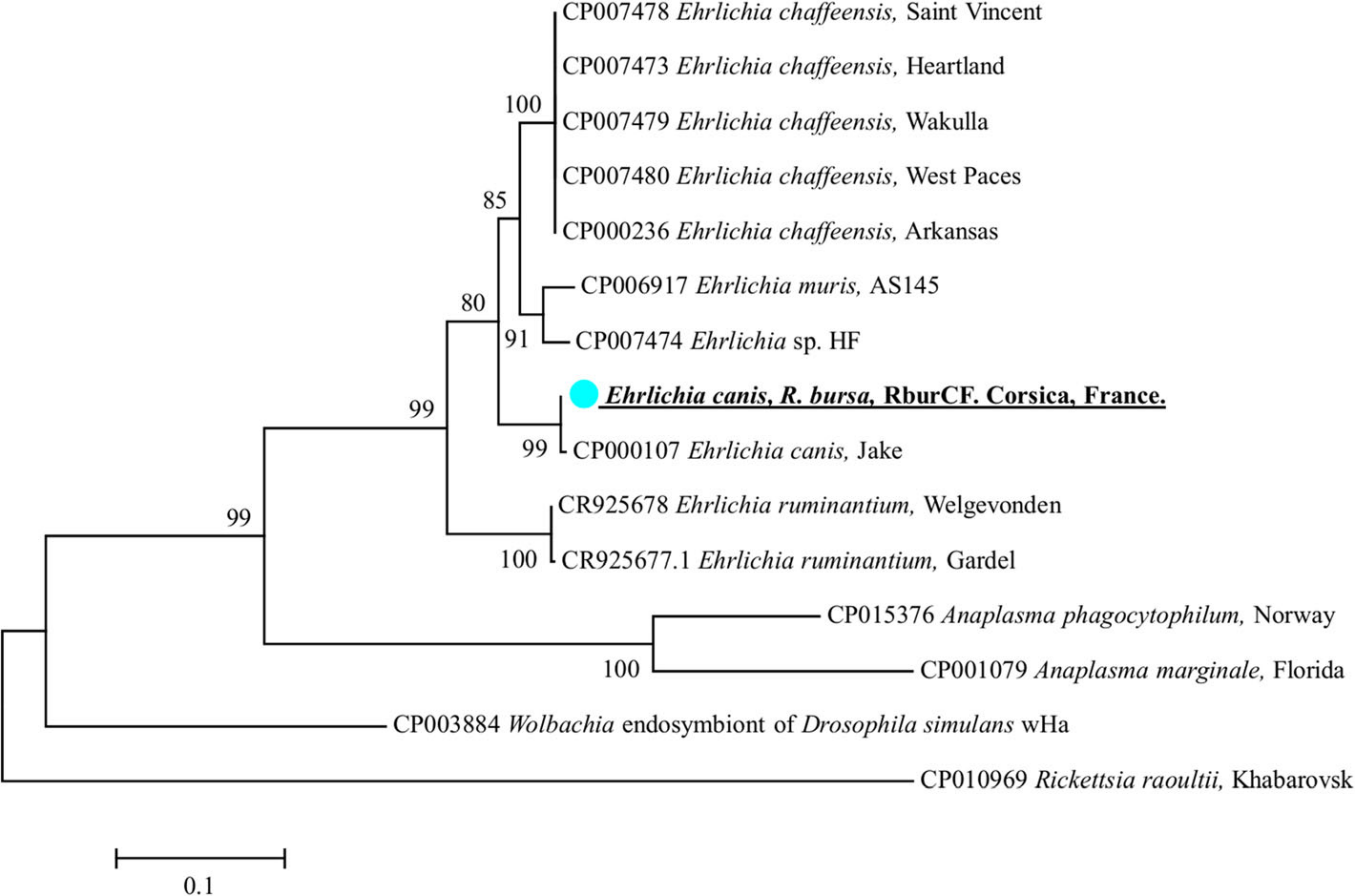
Phylogenetic tree showing the position of *E. canis* amplified from *R. bursa* compared to other *Anaplasmataceae* species. Evolutionary analyses were conducted using MEGA7 [32]. The concatenated 23S rRNA and the *groEl* genes of the *Ehrlichia canis* amplified in this study together with other sequences of *Anaplasmataceae* species available on GenBank. The evolutionary history was. A discrete Gamma distribution was used to model evolutionary rate differences among sites [4 categories (+G, parameter = 0.6567)]. The analysis involved 15 nucleotide sequences. All positions containing gaps and missing data were eliminated. There was a total of 1039 positions in the final dataset. The scale-bar represents a 5% nucleotide sequence divergence

*Anaplasmataceae* DNA was detected in three *R. bursa* of the 123 ticks examined (2.4%). After the 23S rRNA gene sequencing of the *Anaplasmataceae* DNA present in the three ticks, *A. marginale* was identified in two ticks. The two sequences of *A. marginale* were identical to each other and showed 100% homology with the *A. marginale* strain Dawn (CP006847) and Gypsy Plains (CP006846) reported from Australia, and 99% with the *A. marginale* strain Florida (CP001079) and St. Maries (CP000030) reported from the USA (Fig. 3). Finally, based on the 23S rRNA analysis, *E. canis* was identified from the third positive tick*.* These sequences presented 99% homology with the *E. canis* strain Jack (CP000107) reported from the USA (Fig. 4).

### *Anaplasmataceae* species screening from animal blood

The results are summarised in the (Table 3). Of the total of 458 blood samples analysed (Table 1), 32.1% (147) were positive for the initial 23S rRNA qPCR screening. The prevalence of *Anaplasmataceae* infections was as follows: sheep 59.5% (131/220), cattle 100% (12/12) and goats 80% (4/5), whereas all blood samples from horses and dogs were negative. Identification of bacterial species was achieved by amplification followed by sequencing of the portion of the 23S rRNA gene. Seventy-one percent (93/131) of *Anaplasmataceae*-positive sheep samples were infected by *A. ovis*. The 23S sequences obtained were identical to each other and showed 100% identity with *A. ovis* strain KMND Niayes-14 reported in sheep from Senegal [33]. All the other 38 qPCR-positive sheep (29%) were found to be infected by several as yet uncharacterised and potentially new species of *Anaplasma*. In 13/131 (9.9%) of infected sheep, the obtained sequences were identical to each other and showed only 96% identity with the *A. ovis* strain KMND Niayes-14. Due to the absence of additional data on this *Anaplasma* and genetic relatedness to *A. ovis*, we refer to this genotype here as *Anaplasma* cf. *ovis*. There were 3/131 (2.3%) sheep infected by another genotype of *Anaplasma.* These three sequences had 96–98% identity to each other and showed 91–94% identity with the *A. phagocytophilum* strain Norway Variant 2, reported from sheep in Norway (CP015376). We are provisionally calling this incompletely characterised bacterium “*Candidatus* Anaplasma corsicanum”. Finally, a third genotype was found infecting 22/131 (16.8%) sheep, sampled only in 2015. All sequences of this genotype were identical to each other and showed 95% identity with the *A. centrale* strain Israel (CP001759) reported from Israel (Fig. 3). We are provisionally calling this bacterium “*Candidatus* Anaplasma mediterraneum”.

**Table 3:** Overall results and *Anaplasmataceae* species reported in the present study

Species	Sheep	Cattle	Goats	Equine	Dogs	*R. bursa*	*Hy. m. marginatum*
*A. ovis*	93/220 (71%)	0	0	0	0	0	0
*A. marginale*	0	0	0	0	0	2/118 (1.7%)	0
*Anaplasma* cf. *marginale*	0	12/12 (100%)	4/5 (80%)	0	0	0	0
*Anaplasma* cf. *ovis*	13/220 (9.9%)	0	0	0	0	0	0
“*Canditatus* Anaplasma corsicanum”	3/220 (2.3%)	0	0	0	0	0	0
“*Candidatus* Anaplasma mediterraneum”	22/220 (16.8%)	0	0	0	0	0	0
*E. canis*	0	0	0	0	0	1/118 (0.8%)	0
Totals	131/220 (59.5%)	12/12 (100%)	4/5 (80%)	0	0	3/118 (2.5%)	0

Data presented as No. of infected/No. of examined (Prevalence %)

All 12 cattle tested were positive in qPCR and conventional PCR (100%) for *Anaplasmataceae* bacteria. Sequencing analyses showed that all cattle were infected by *A. marginale*. The sequences were identical to each other, and also to the sequences of *A. marginale* identified in the *R. bursa* ticks removed from the same animals.

Finally, 4 of 5 goats were found to be infected by a potentially new species of *Anaplasma* similar to *A. marginale*. All sequences were identical to each other and showed 99% homology with the *A. marginale* strain Dawn (CP006847), *A. centrale* strain Israel (CP001759) and 99% with *A. ovis* strain KMND Niayas-14 (KM021411) (Fig. 3).

Additional characterisation of detected *Anaplasmataceae* bacteria was performed by amplification/sequencing of a portion of the *rpoB* gene (for *Anaplasma*-positive samples) or *groEL* gene (for *Ehrlichia*-positive samples). *RpoB* sequences from *A. ovis*-positive samples were also identical to each other and showed 100% identity with *A. ovis* strain KMND Nayes-14. *rpoB* sequences from two *A. marginale-*positive *R. bursa* ticks and the four other sequences obtained from cattle blood samples were identical to each other and showed 100% identity with *A. marginale* strain Dawn (CP006847) and Gypsy Plains (CP006846) and 99% with *A. marginale* strain Florida (CP001079) and St. Maries (CP000030). For the *E. canis* identified in one *R. bursa* tick, the DNA sample was amplified using *groEl Ehrlichia* genus-specific primers and sequenced. The sequence showed 99% homology with the *E. canis* strain Jack (CP000107) (Fig. 4).

Analysis of *rpoB* sequences of all three novel genotypes of *Anaplasma* produced results similar to the 23S gene analysis. *RpoB* sequences from *A.* cf. *ovis* showed 98% identity with *A. ovis* strain KMND Nayes-14. The three *rpoB* sequences from “*Ca*. Anaplasma corsicanum” had 99% identity to each other, and only 80% with *A. phagocytophilum* strains Norway Variant 2 (CP015376), Dog2 (CP006618), JM (CP006617) and HZ (CP000235). *RpoB* sequences of “*Ca*. Anaplasma mediterraneum” presented 84% identity with *A. centrale* strain Israel (CP001759). Finally, *Anaplasma* cf. *marginale* from four goats had *rpoB* sequences that shared 98% identity with *A. ovis* strain KMND Niayes-14 (KX155494) and strain RhburBas11 (KX155495), 93% with *A. marginale* strain Florida (CP001079), St. Maries (CP000030), 89% strain Dawn (CP006847) and Gypsy Plains (CP006846), and 87% with *A. centrale* strain Israel (CP001759) (Fig. 3).

### Phylogenetic analyses of the potentially new species

The phylogenetic tree inferred from the *Anaplasmataceae* concatenated 23S rRNA, and the *rpoB* genes provide evidence that “*Ca*. Anaplasma corsicanum”, *Anaplasma* cf. *ovis*, “*Ca*. Anaplasma mediterraneum” from sheep and *Anaplasma* cf. *marginale* from goats could potentially be new species. *“* *Ca*. Anaplasma corsicanum” clustered separately from the recognised species *A. phagocytophilum*, *A. platys*, *A. ovis*, *A. marginale* and *A. centrale* (Fig. 2)*.* The sequence of *Anaplasma* cf. *ovis* from sheep and the sequence of *Anaplasma* cf. *marginale* from goats clustered together with the sequence of *A. ovis* strain KMND Niayes-14 from Senegal and *A. ovis* from sheep identified in this study with high bootstrap values and separately from the cluster of *A. marginale* species. Finally, the sequence of “*Ca*. Anaplasma mediterraneum” obtained from sheep form well-defined branches with high bootstrap values (93–95%) (Fig. 3).

All sequences obtained in the present study were submitted to GenBank under the following accession numbers: (i) for the 23S rRNA gene: *Anaplasma ovis* OVCF02 (KY498325), *Anaplasma* cf. *ovis* OVCF115 (KY498326), “*Ca*. Anaplasma corsicanum” OVCF72 (KY498327), “*Ca*. Anaplasma corsicanum” OVCF81 (KY498328), “*Ca*. Anaplasma corsicanum” OVCF65 (KY498329), “*Ca*. Anaplasma mediterraneum” OVCFO215 (KY498330), *Anaplasma* cf. *marginale* CpCF01 (KY498331), *Anaplasma marginale* BvCF13 (KY498332), *A. marginale* Rh.burCF08 (KY498334), *A. marginale* Rh.burCF10 (KY498335), *Ehrlichia canis* Rh.burCF07 (KY498333); (ii) for the *rpoB* gene: *Anaplasma ovis* OVCF02 (KY498325), *Anaplasma* cf. *ovis* OVCF115 (KY498336), “*Ca*. Anaplasma corsicanum” OVCF72 (KY498338), “*Ca*. Anaplasma corsicanum” OVCF81 (KY498339), “*Ca*. Anaplasma corsicanum” OVCF65 (KY498340), “*Ca*. Anaplasma mediterraneum” OVCFO215 (KY498341), *Anaplasma* cf. *marginale* CpCF01 (KY498342), *Anaplasma marginale* BvCF13 (KY498343), *A. marginale* Rh.burCF08 (KY498344), *A. marginale* Rh.burCF10 (KY498345); (iii) For the *groEl* gene: *Ehrlichia canis* Rh.burCF07 (KY498324). For tick species, the 12S rRNA sequences were submitted under the following accession number: *Hy. m. marginatum* (KY595783) and *R. bursa* (KY595784).

## Discussion

Livestock farming in Corsica is an important economic activity involving approximately 150,000 sheep, 48,000 goats, 40,000 pigs and 70,000 cattle [22]. The significance of anaplasmosis in animals in Corsica is not yet known. *Anaplasma* infection may likely be neglected because of its unknown economic importance in small ruminants. To our knowledge, the present study is the first report of the incidence of *Anaplasmataceae* species in ticks and animals in Corsica. Furthermore, the presence and molecular traits of six species belonging to the genus *Anaplasma* from ruminants and ticks infecting cattle, and one *Ehrlichia*, are shown. The typical Mediterranean environment of Corsica with hot summers, along with the geographical location, favours the spread of seasonal tick infestations. Two tick species were collected and confirmed by the morphological and molecular investigation as *R. bursa* and *Hy. m. marginatum* (Fig. 1). Neither the tick fauna of Corsica nor the transmitted pathogens have been fully investigated. Here, ticks were only collected from cattle; infestation of other animals, including sheep, goats, horses and dogs, was not observed. A previous study demonstrated the presence of three species of the genus *Hyalomma* in Corsica: *Hy. marginatum*, *Hy. aegyptium* and *Hy. rufipes* [22]. While *Hy. marginatum* is found on many hosts, *Hy. aegyptium* was identified once in Corsica on a *Testudo hermanni* tortoise, while *Hy. rufipes* has been collected from migrating birds [22]. Recently, *Hy. scupense* was also identified and collected from Corsican cattle by Grech-Angelini et al. [22]. *Rhipicephalus bursa* was the most common tick infesting cattle in our study. This two-host species occurs in the entire Mediterranean, Adriatic and Aegean basins, including their islands, and North Africa [25, 34]. *Rhipicephalus bursa* prefers grassy slopes and low to medium altitude mountain slopes, as well as certain modified steppe and semi-desert environments [35]. However, this tick species is recorded in cold regions, including the Atlantic region of Europe, the French Basque country, Spanish Basque country, and north-west Portugal [28, 35]. Corsica is a typical Mediterranean ecosystem, which favours the spread of these ticks. *Rhipicephalus bursa* mature and adults infest many hosts, including cattle, sheep, goats and other domestic animals, whereas wild ungulates are the original host [36]. This species is a recognised vector of many pathogens, including *Babesia ovis*, *Theileria* spp., *A. marginale* and *A. ovis* [36]. DNA of *Coxiella burnetii* and *A. phagocytophilum* have also been amplified from these ticks [28, 35]. Here, the DNA of *A. marginale* was amplified from two engorged female ticks removed from cattle infected by *A. marginale.* Previous studies have reported *A. marginale* from *R. bursa* removed from cattle in Portugal [34], and from Iberian red deer and European wild boar in Spain [37]. It is likely that the presence of *A. marginale* DNA in these two ticks was due to the presence of this pathogen in the blood meal. However, the percentage of *R. bursa*-engorged females in our study was 42.9% (30/70 female); only two engorged ticks were found to harbour *A. marginale*.

*Ehrlichia canis* was amplified from one non-engorged *R. bursa* female. In Europe, *E. canis* is associated with the presence of the brown dog tick *R. sanguineus* [38]. However, in the Mediterranean area, *E. canis* has also been reported from *R. bursa* collected from goats in Sardinia, Italy [39] and *Cediopsylla inaequalis* collected from red foxes in Sicily, Italy [40]. In other European countries, there are reports of *E. canis* from *D. marginatus* collected from dogs, *Ixodes canisuga* collected from red foxes, and *I. ricinus* collected from vegetation in Hungary [41, 42]. Domestic animals are now recognised as the primary hosts of *R. bursa* [36]; however, the role of *R. bursa* and the other arthropod species in the transmission of *E. canis* remains unknown.

None of the five *Hy. m. marginatum* ticks were positive for *Anaplasmataceae* infection. However, in Spain, *Hy. m. marginatum* has been identified as a potential biological vector for *A. marginale* [43]. These ticks are also the vectors of *Babesia caballi*, causing babesiosis in horses and *Theileria annulata* infection under laboratory conditions [26]. Other studies are needed to clarify and list the pathogens associated with these ticks in Corsica.

The prevalence of *Anaplasma* spp. in our study was surprisingly high in ruminants. Based on the 23S rRNA gene molecular investigations, the individual prevalence observed was 59.5% in sheep, 100% in cattle, and 80% in goats. However, none of the canine or equine blood samples was positive. Genetic characterisation using 23S rRNA and the *rpoB* genes identified *A. ovis*, *A. marginale*, and several potentially new species, all belonging to the genus *Anaplasma*. These data confirm the relevance of ruminants as important hosts and reservoirs of different *Anaplasma* species in the Mediterranean ecosystem. The prevalence of *Anaplasma* spp. in ruminants examined by us was lower than the prevalence data reported from Sardinia [44]. The prevalence of *A. marginale* infection in cattle was higher than that observed in cattle in Sicily [45]; however, in that study, the number of samples analysed was greater than in our study. In sheep, the prevalence reported in our study was lower than that reported in Sicily [46].

Sheep, goats, and cattle sampled in this study manifested poor health. In sheep, most clinical manifestations observed were relapsing fever, drop in milk production and mortality. Molecular and phylogenetic analysis of sequences amplified from sheep blood samples were identified *A. ovis*, and three potentially new species, “*Ca*. Anaplasma corsicanum”, “*Ca*. Anaplasma mediterraneum”, and *Anaplasma* cf. *ovis*. In the Mediterranean area, *A. ovis* is reported to be endemic to Sicily [47, 48]. This pathogen has also been reported from Greece and Cyprus [21, 49]. In Europe, *A. ovis* has also been reported from Portugal, Hungary [19] and Slovakia [47]. *Anaplasma ovis* infection in the mouflon and the European roe deer has been reported from Cyprus and southern Spain, respectively [50, 51]. The main vector of *A. ovis* in Europe is *R. bursa* [28]. However, *A. ovis* DNA was amplified from *I. ricinus* removed from cattle in Hungary [15], *Haemaphysalis sulcata* removed from mouflons in Cyprus [50], and the sheep ked (*Melophagus ovinus*) and deer ked (*Lipoptena cervi*) in Hungary [48]. In addition, in provinces of Palermo and Ragusa (Italy), *A. ovis* was amplified from foxes, and a flea, *Xenopsylla cheopis*, removed from these foxes [40]. The role of these arthropods and insects in the transmission of *A. ovis* remains unclear. Anaplasmosis in sheep is usually subclinical. This bacterium can lead to severe infection with severe illness in sheep; severe illness can occur in some extreme conditions, such as the association with more than one parasitic disease or other stress factors [49, 52].

All cattle sampled in this study were infected with *A. marginale*. In this farm, the farmer reported mortality in his livestock. Anaemia and icterus were most observed in other cattle (Table 1). Bovine anaplasmosis due to *A. marginale* causes mild to severe anaemia, icterus, fever, weight loss, abortion and lethargy [53]. In Europe, *A. marginale* is mainly present in the Mediterranean region, alpine, and eastern areas [16]. In the Mediterranean region, the DNA of this bacterium has been amplified from *D. reticulatus*, *D. marginatus*, *R. turanicus*, *Haemaphysalis punctata*, *Hy. m. marginatum* and *R. bursa* [34, 37, 43]. Interestingly, *A. marginale* has been amplified from *Xenopsylla cheopis* removed from red foxes in Italy [40]. Outside of the Mediterranean region, *A. marginale* has been amplified from *I. ricinus* and *Tabanus bovis* in Hungary [15, 43]. The role of *I. ricinus*, *Tabanus bovis* and *Xenopsylla cheopis* in the transmission of *A. marginale* remains unclear.

The potentially new species “*Ca*. Anaplasma corsicanum” and “*Ca*. Anaplasma mediterraneum” have genetic features which are different from other species of the genus *Anaplasma* (Fig. 3). Phylogenetic analysis based on the concatenated 23S rRNA and *rpoB* genes showed that “*Ca*. Anaplasma corsicanum” is related to *A. phagocytophilum*, but clustered separately from recognised species. “*Ca*. Anaplasma mediterraneum” is related to *A. centrale* and forms a distinct subcluster. Two other identified genotypes, *Anaplasma* cf. *ovis* and *Anaplasma* cf. *marginale,* grouped with the sequences of *A. ovis* (Fig. 3). Interestingly, despite this grouping, *Anaplasma* cf. *marginale* is closer to *A. marginale* than to *A. ovis*, based on the 23S rRNA comparison. The *rpoB* encodes the RNA polymerase subunit beta and gives a better statistical score for differentiating between the closest species of *Anaplasma* spp., with more sequence variations [28]. The observed prevalence of the potentially new *Anaplasma* species in sheep was low (17.3%, 38/220) compared to the prevalence of *A. ovis*; however, 80% (4/5) of the goats sampled in this study were infected by *Anaplasma* cf. *marginale*. The importance of this amplified *Anaplasma* species remains to be understood.

Mortalities in animals were reported by the farmers in the sheep and cattle herds. Unfortunately, we did not have access to body tissue of fluid from the dead animals to perform a post-mortem diagnosis. The different reported symptoms and the results found in the present study with the high prevalence of *A. marginale* in cattle and *A. ovis* and the others amplified *Anaplasma* spp. in sheep and goats suggest that the mortalities can be linked to these *Anaplasma* species. However, other tick- or vector-borne diseases can also lead to mortalities like Piroplasmosis [54, 55]. In addition, co-infection by two or more pathogens can lead to increase the pathogenicity and clinical manifestations in animals and resultant varying outcomes on host health and survival [56]. The involvement or not involvement of the *Anaplasmataceae* species amplified in the present study should be considered with caution do to the possible implication of other pathogens.

In our study, we did not find *A. phagocytophilum* in animal or tick samples. *Anaplasma platys* and *E. canis* were also not found in dogs, although *E. canis* was found in *Rh. bursa* collected from a cow.

## Conclusion

The present study demonstrates that ruminants in Corsica are a reservoir for multiple *Anaplasma* species, whereas *R. bursa* seems to be a vector of *A. marginale* in cattle. The prevalence of *Anaplasma* spp. infection was high. The use of quantitative real-time PCR complemented with sequencing and genetic characterisation using two genes, *rpoB* and *groEl*, revealed an interesting diversity of *Anaplasma* spp. infection in small ruminants and *R. bursa*, including potentially new species and *E. canis* in one *R. bursa* tick. Nevertheless, characterisation studies are needed to ascertain the pathogenesis and/or the zoonotic potential of the strains and their significance for animals and public health.

## Abbreviations

*GroEl* gene: Heat shock protein gene
qPCR: Quantitative real-time polymerase chain reaction
*RpoB* gene: RNA polymerase subunit beta gene
